# 2,5-Dimercapto-1,3,4-thiadiazole-modified gel electrolyte for reduced shuttle effect and enhanced redox kinetics of lithium–sulfur batteries

**DOI:** 10.1039/d5ra06093a

**Published:** 2025-10-27

**Authors:** Xiangzhe Lin, Junlin Wang, Xu Tang, Manru Yang, Nairong Chen, Feng Li, Fengxiang Zhang

**Affiliations:** a Leicester International Institute, Dalian University of Technology Dalian 124221 China linxiangzhe25fz@163.com; b School of Chemical Engineering, Ocean and Life Sciences (State Key Laboratory of Fine Chemicals), Dalian University of Technology Dalian 124221 China zhangfx@dlut.edu.cn; c College of Material Engineering, Fujian Agriculture and Forestry University Fuzhou 350002 China fengli@fafu.edu.cn

## Abstract

The safety risks of liquid electrolyte leakage in conventional batteries have prompted intensive research on solid electrolytes for enhanced safety and energy density. Here, we report a novel PVDF gel electrolyte (GE) by incorporating 2,5-dimercapto-1,3,4-thiadiazole (DMcT). By virtue of the active thiol groups (S–H), DMcT can function as a difunctional mediator to suppress the shuttle effect and accelerate the redox kinetics of Li–S batteries, thereby enabling boosted rate performances and enhanced cycling stability of Li–S batteries. At 1C, the Li–S battery retains 180.5 mA h g^−1^, outperforming control cells. At 0.5C, it exhibits an ultralow decay rate (0.13% per cycle) with 99% coulombic efficiency. This work presents a novel strategy to address the shuttle effect and improve the rate performance in gel electrolyte Li–S batteries, paving the way for safer high-energy battery systems.

## Abstract 2

A difunctional mediator is incorporated into a PVDF GE to suppress the shuttle effect and ac, celerate the redox kinetics of Li–S batteries.

## Graphical abstract

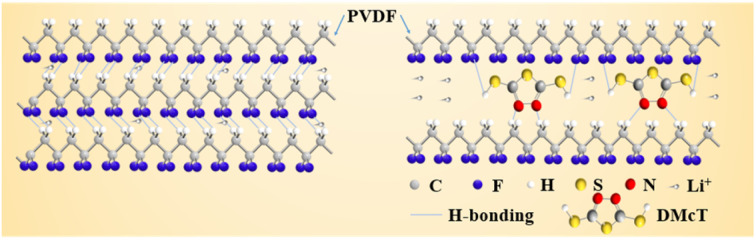

## Introduction

Lithium–sulfur (Li–S) batteries are recognized as promising candidates for next-generation storage systems, benefiting from their high energy density (2500 Wh kg^−1^) and low cost.^1–3^ Despite these merits, Li–S batteries face some significant challenges in practical applications, especially the “shuttle effect” of soluble lithium polysulfides (LiPSs) which leads to active sulfur deficiency, rapid capacity decay, and short cycling lifespan.^4,5^ This effect mainly results from the fact that LiPSs tend to dissolve in liquid electrolytes (LEs) during discharge/charge processes and thus migrate to the Li anode.^6^ As the traditional electrolyte, LEs are also prone to leakage and flammability, which cause safety problems.^7^ By contrast, solid electrolytes (SEs) can effectively eliminate the above problems due to their structural integrity and stability. However, SEs are subjected to an interface mismatch that limits the utilization of sulfur with low energy density and causes uncontrollable Li dendritic growth on the anode.^8–13^ Gel electrolytes (GEs), as an intermediate state between the liquid and solid, are formed by incorporating liquid electrolytes in a polymer matrix. GEs not only reserve the high ionic conductivity and sufficient electrode–electrolyte compatibility of liquid electrolytes but also offer the benign safety and structural stability of solid electrolytes.^14–16^ Thus, GEs are regarded as a promising alternative for the development of high-performance Li–S batteries.

GEs based on poly (vinylidene fluoride) (PVDF) have gained considerable attention in Li–S batteries due to their excellent chemical stability, mechanical strength, and processability. However, PVDF-based GEs exhibit low ionic conductivity and weak polysulfide blocking capability, as imposed by their highly crystalline network and the lack of interacting sites with LiPSs. To address these issues, several strategies have been proposed for improving the electrochemical performances of PVDF GE-assembled Li–S batteries. For example, poly (vinylidene fluoride difluoro-hexafluoropropylene) (PVDF-HFP), a copolymer of PVDF, is designed to lower the crystallinity for higher LE absorption and Li^+^ migration, thereby enhancing the ionic conductivity. Meanwhile, the decreased crystallinity leads to low mechanical strength that is inadequate to restrict the growth of Li dendrites and polysulfide shuttling. Hence, organic polysulfide polymer (PSPEG^17^) and inorganic nanofillers (*e.g.*, Ti_32_O_16_,^18^ Li_1.5_Al_0.5_Ti_1.5_(PO_4_)_3_)^19^ have been blended into the PVDF system to inhibit the shuttle effect, restrict the growth of lithium dendrites, and increase the ionic conductivity, but they either require complex preparation or fail to be applied at high current densities due to the low redox kinetics. To sum up, despite great efforts, developing PVDF GEs with remarkable mechanical robustness, excellent ionic conductivity, reduced shuttle effect, and enhanced redox kinetics for the application of Li–S batteries remains a significant challenge.

Herein, 2,5-dimercapto-1,3,4-thiadiazole (DMcT), a heterocyclic compound with two reactive thiol groups (-SH), is incorporated to reduce the crystallinity of PVDF for enhanced ionic conductivity while improving the mechanical properties of PVDF GE for structural stability. More notably, DMcT can serve as a difunctional mediator in PVDF GE. On the one hand, DMcT functions as an absorbing mediator that facilitates the adsorption of polysulfides to suppress the shuttle effect by virtue of its –SH groups. On the other hand, DMcT acts as a redox mediator that lowers activation energy for higher redox kinetics. Owing to the efficacy of DMcT, the Li–S batteries assembled with PVDF-DMcT have been demonstrated to display excellent rate performance that retains a specific capacity of 180.5 mA h g^−1^ at 1C, as well as long-cycle stability with an ultra-low-capacity decay rate of 0.13% per cycle and a high coulombic efficiency of 99.5% after 300 cycles. This strategy offers a feasible way to establish high-performance electrolytes for Li–S batteries.

## Materials and methods

### Materials

2,5-Dimercapto-1,3,4-thiadiazole (97%) and *N*,*N*-dimethylformamide (DMF, AR) were purchased from Shanghai Aladdin Biochemical Technology Co., Ltd. Polyvinylidene fluoride (PVDF, HSV900) was obtained from Arkema Co., Ltd.

### Preparation of gel electrolyte (GE)

The PVDF-DMcT GE was prepared by the following steps. At first, 4.8 g PVDF and 1.2 g DMcT were dissolved in 60 mL DMF and stirred at 80 °C to obtain the PVDF-DMcT solution. Then, the PVDF-DMcT solution was poured onto the glass plate, and the solvent was evaporated to obtain the PVDF-DMcT film. The membrane with a 16 mm diameter was then immersed in a mixed solution of 1,3-dioxolane (DOL) and ethylene glycol dimethyl ether (DME) containing 2 wt% LiNO_3_ and 1 M lithium trifluoromethylsulfonimide (LiTFSI) (VDOL : VDME = 1 : 1) for 12 h to finally obtain PVDF-DMcT GE (0.130 mm thickness). The pure PVDF GE was also prepared under the same conditions for comparison.

### Preparation of the sulfur cathode and assembly of the lithium sulfur battery

The sulfur powder and conductive carbon black (BP2000) were mixed with a mass ratio of 3 : 1 and heated at 155 °C for 12 h in a vacuum box to obtain the carbon sulfur composite (BP2000/S). Then, BP2000/S, super P, and PVDF were mixed with a mass ratio of 7 : 2 : 1 and dispersed in the *N*-methyl pyrrolidone (NMP) solvent to prepare the cathode slurry. The fabricated slurries were coated onto an aluminum foil, and the dried aluminum foil was cut into discs with a diameter of 12 mm to obtain a cathode electrode. The average sulfur loading of the sulfur electrode is approximately 1.5 mg cm^−2^. The obtained carbon/sulfur cathode electrode, gel electrolyte, and lithium anode electrode were assembled into a CR2016 button battery in a glove box filled with argon. Before the battery test, it was kept in a 30 °C incubator for 8 h.

### Characterization of materials

Fourier transform infrared spectrometer (FTIR) was employed with a Nicolet380 FTIR spectrometer at a resolution of 4 cm^−1^ and 32 scans with the test range from 400 to 4000 cm^−1^. X-ray diffraction (XRD) patterns were conducted on Lab XRD-7000 s with a 2*θ* range of 10–80°. X-ray photoelectron spectroscopy (XPS) was performed on the ESCALAB250Xi spectrophotometer instrument. The morphology of the samples was investigated using Nano SEM 450 Nova scanning electron microscopy (SEM). UV-vis absorption spectra were recorded with a Hitachi UH5300 spectrometer to detect the concentration of polysulfides.

The liquid absorption rate (*η*) is the amount of liquid electrolyte absorbed by the GE membrane at room temperature. First, the GE film was weighed (marked as *m*_0_), placed in the liquid electrolyte for 2 h, wiped with paper and weighed again (marked as *m*_1_). The liquid absorption rate (*η*) of the GE membrane is calculated according to formula (1):1
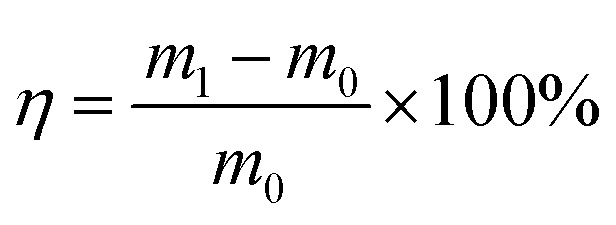


The tensile strength and elongation at break of PVDF and PVDF-DMcT films after absorbing the liquid electrolyte were measured using an electromechanical Universal Testing Machine (ETM) 102A.

### Electrochemical performance test of the GE film

CR 2016-type coin cells were assembled in a glove box (S-super 1220/750/900)filled with argon gas. Cyclic voltammetry (CV) tests were performed on an electrochemical workstation (CEI760E). Taking the open circuit voltage as the starting voltage, the Li–S battery was tested in the voltage range of 1.7–2.8 V, and the sweep speed was 0.1–0.3 mV s^−1^. Lithium-ion diffusion coefficient (*D*_Li^+^_) can be calculated by the CV tests.

2 *I*_p_ = (2.69 × 10^5^)^1.5^_*n*_*SD*_Li^+^_^0.5^*C*_Li_*v*^0.5^

In eqn (2), *I*_p_, *n*, *S*, *v* and *C*_Li_ represent the peak current, charge transfer number of different redox peaks, electrode area, cyclic voltammetry scanning speed and Li^+^ concentration in the electrolyte, respectively.

The Li^+^ transference number (*t*_Li^+^_) can be calculated according to eqn (3):3
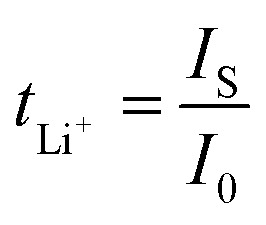
In eqn (3), *I*_0_ and *I*_S_ represent the currents at the initial and steady states, respectively.

Electrochemical impedance spectroscopy (EIS) tests of the assembled coin cells were performed in an electrochemical workstation (CEI760E). Taking the open circuit voltage as the starting voltage, the measured frequency range is 0.01–10^5^ Hz, and the amplitude is 5 mV. The rate and cycle performance test of the assembled coin cells was conducted in a Land CT3001A system. After standing for 6–8 h, the assembled conventional Li–S battery was tested at different current densities (1C = 1675 mA h g^−1^) in the voltage range of 1.7–2.8 V, and the charge–discharge cycle was tested at constant current density.

## Results and discussion

### Morphology and structural characterization

**Fig. 1:**
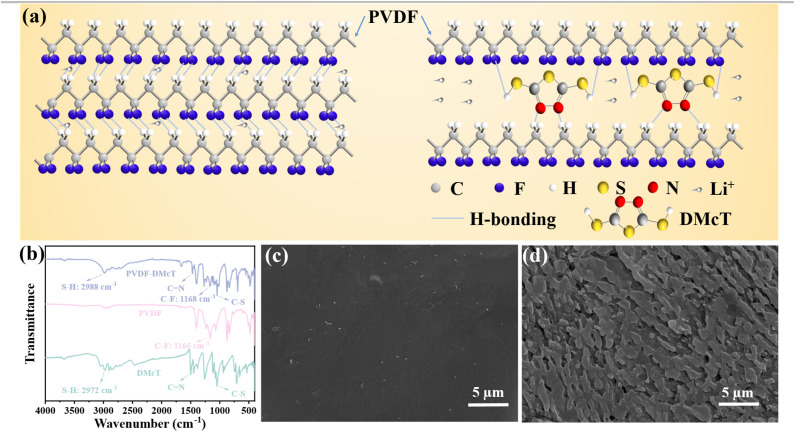
(a) Structural design of PVDF-DMcT GE in Li–S batteries. (b) FTIR spectra of DMcT, PVDF, and the PVDF-DMcT composite. SEM images of the (c) PVDF and (d) PVDF-DMcT samples.

Fig. 1a shows the structural design of PVDF-DMcT GE in Li–S batteries that harnesses DMcT as a difunctional mediator to simultaneously enhance polysulfide adsorption and catalyse lithium-ion transference of the Li–S battery system. In general, PVDF molecules tend to crystallize *via* the generation of intermolecular hydrogen bonds, thus hindering the transport of Li^+^. After importing DMcT based on a facile one-step method, the crystalline structure of PVDF is partially damaged through the formation of S–H⋯F–C hydrogen bonds between DMcT and PVDF, as evidenced by the FTIR results showing the peak shift of the –SH group (S–H stretch, 2972 → 2988 cm^−1^) and –CF bond (C–F stretch, 1166 → 1168 cm^−1^) in the PVDF-DMcT sample (Fig. 1b).^20,21^ The less crystalline structure of the PVDF-DMcT GE can also be verified by XRD patterns that show the obvious decrease of characteristic PVDF peaks at 36.9° and 39.5° (Fig. S1). SEM images further demonstrate the structural change of the PVDF-DMcT sample. As observed in Fig. 1c and d, the pure PVDF sample presents a dense structure that impedes the migration of Li^+^. Introducing DMcT enables PVDF to display a lamellar and porous network, which provides continuous conduction pathways for Li^+^. Besides, such a structure allows PVDF-DMcT GE with a higher absorption rate of LEs (67.58%) (Fig. S2 and S3). It is remarkable that, although the looser structure is formed, the PVDF-DMcT sample containing 20 wt% DMcT (named as PVDF-20%DMcT) exhibits much-improved mechanical properties compared to that of the PVDF counterpart, which may be attributed to the versatile reversible bonds between the PVDF and the DMcT (Fig. S4). In view of the optimal mechanical properties, PVDF-20%DMcT GE is selected for the following experiments.

**Fig. 2:**
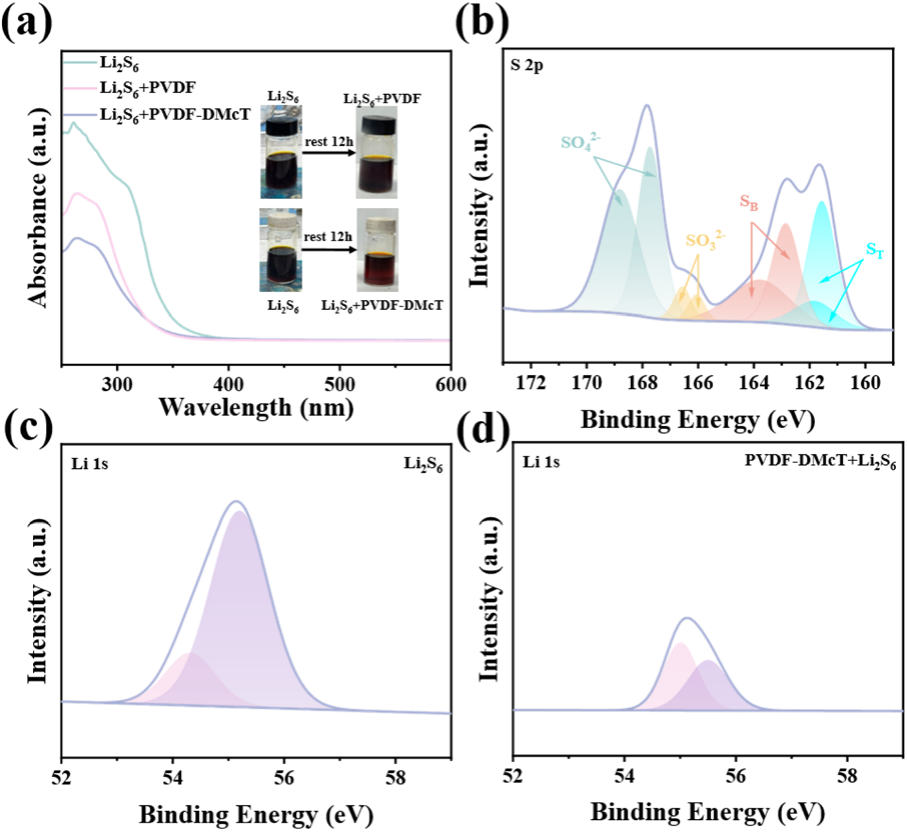
(a) UV-vis spectra and photographs (inset) of the Li_2_S_6_ solution before and after placing the PVDF and PVDF-DMcT GEs for 12 h. (b) S 2p XPS spectra of PVDF-DMcT GE after immersing in Li_2_S_6_ solution for 12 h. (c and d) Li 1 s XPS spectra of the Li_2_S_6_ solution without and with PVDF-DMcT GE.

In addition to facilitating Li^+^ transport, DMcT also plays a critical role in enhancing the polysulfide adsorption of PVDF GE. As revealed in Fig. 2a, compared to the Li_2_S_6_ solution containing PVDF, the Li_2_S_6_ solution containing PVDF-DMcT showcases significantly lighter color after setting for 12 h, suggesting the effective polysulfide adsorption derived from DMcT. This finding is in accordance with the results of the UV-vis spectra of PVDF-DMcT, which show the significant decrease of Li_2_S_6_ peaks at 260–280 nm with an extended setting time. XPS measurements further indicate the polysulfide adsorption capability of DMcT, as evidenced by the existence of the characteristic terminal S (161.6/161.9 eV) and bridging S (162.9/163.8 eV) peaks (Fig. 2b) presented in the S 2p spectra, and the decreased intensity of Li_2_S_6_ peaks (54.3 and 55.2 eV) shown in the Li 1 s spectra (Fig. 2c and d). The ability of DMcT to effectively absorb the polysulfide discloses the potential of PVDF-DMcT GE on mitigating the shuttle effect in Li–S batteries.

### Electrochemical performance of PVDF-DMcT

**Fig. 3:**
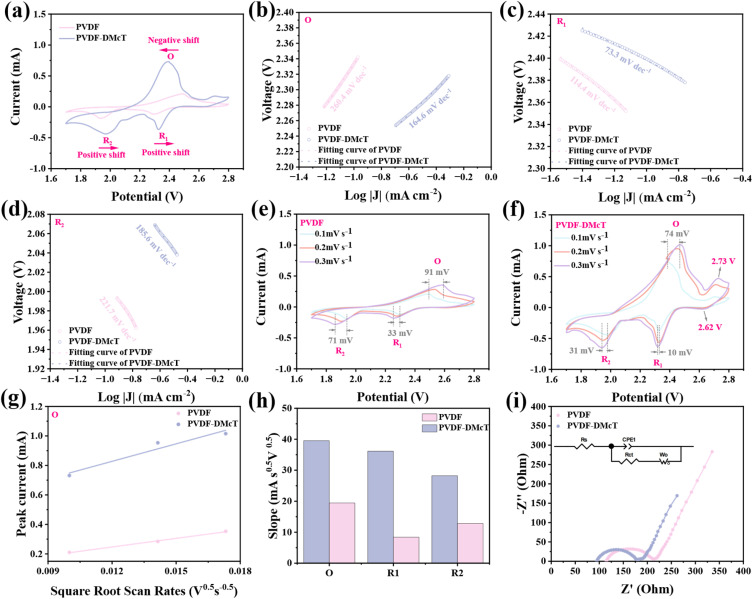
(a) CV curves of Li–S batteries with PVDF and PVDF-DMcT GEs at a scan rate of 0.1 mV s^−1^. (b–d) Tafel plots for the O, *R*_1_, and *R*_2_ peak. (e and f) CV curves of Li–S batteries with PVDF and PVDF-DMcT GEs at different scanning rates. (g) Fitting plot of peak current at the O peak *versus* the square root of the scan rate. (h) Slope values of *I*_p_/*γ*^0.5^ for the Li–S batteries with PVDF and PVDF-DMcT GEs. (i) EIS plots of Li–S batteries with PVDF and PVDF-DMcT GEs.

We then evaluate the electrochemical behavior of the PVDF-DMcT GE *via* cyclic voltammetry (CV). As shown in Fig. 3a, compared to the Li–S battery with PVDF GE, the battery with PVDF-DMcT GE exhibits higher peak currents, as well as the shift of oxidation peak for Li_2_S → S_8_ (O, 2.49 → 2.39 V) and reduction peaks for S_8_ → Li_2_S_*n*_ (*R*_1_, 2.29 → 2.33 V) and Li_2_S_*n*_ → Li_2_S (*R*_2_, 1.93 → 1.97 V). These results confirm that DMcT improves the redox kinetics of Li–S batteries, which may contribute to suppressing polysulfide dissolution and reducing polarization. The Tafel slope is then analysed to further elucidate the effect of DMcT on PVDF-DMcT GE. As illustrated in Fig. 3b–d, the slope of the redox reaction based on PVDF-DMcT GE is smaller than that of PVDF GE, implying that DMcT can catalyze redox reactions by lowering the energy barriers for Li_2_S conversion.

The effectiveness of DMcT on improving the redox kinetics of Li–S batteries is also confirmed by CV tests at different scan rates. As displayed in Fig. 3e and f, the Li–S battery with PVDF-DMcT GE exhibits a smaller redox peak shift compared to that with PVDF GE, indicating higher reversibility and less polarization. Additionally, the Li–S battery with PVDF-DMcT GE appears to be a reversible redox couple at 2.62/2.73 V (Fig. 3f). The reduction peak at 2.62 V may correspond to the process of S radicals formed by the cleavage of S–H bonds in DMcT. Also, the oxidation peak at 2.73 V may be associated with the formation of DMcT and S element during the process of removing lithium from DMcT LiS_*n*_ (4 ≤ *n* ≤ 8).^22^ The mass transfer behavior and reversibility of PVDF-DMcT GE are further investigated by plotting the peak current density (*I*_p_) of cathodic reactions as a function of the square root of the scan rate (*v*_0.5_) (Fig. 3g, h, S5a and b). In PVDF-DMcT GE, the slopes of the linear variation of *I*_p_ with respect to *v*_0.5_ during the oxidation and reduction processes are higher than those of PVDF GE. Based on eqn (2) (electron transfer number of oxidation peak *n*_O_ = 2, electron transfer number of reduction peak 1 *n*_R_1__ = 0.5, electron transfer number of reduction peak 2 *n*_R_2__ = 1.5, electrode area *S* = 1.13 cm^2^, and lithium-ion concentration 10^−3^ mol mL^−1^), the lithium-ion diffusion coefficients (D_Li^+^_) for PVDF-DMcT and PVDF were calculated as 2.11 × 10^−9^ cm^2^ s^−1^ and 5.10 × 10^−10^ cm^2^ s^−1^, respectively, at the oxidation peak. The lithium-ion diffusion coefficients (D_Li^+^_) for PVDF-DMcT and PVDF were calculated as 1.13 × 10^−7^ cm^2^ s^−1^ and 6.10 × 10^−9^ cm^2^ s^−1^, respectively, at reduction peak 1. The lithium-ion diffusion coefficients (D_Li^+^_) for PVDF-DMcT and PVDF were calculated as 2.55 × 10^−9^ cm^2^ s^−1^ and 5.27 × 10^−10^ cm^2^ s^−1^, respectively, at reduction peak 2. This observation implies the enhanced Li^+^ diffusion coefficient, which is attributed to the reduced crystallinity of PVDF for more transport channels and the catalytic effect derived from DMcT for lower activation energy. Additionally, the electrochemical impedance spectroscopy (EIS) curve shows that the PVDF-DMcT-based Li–S battery exhibits lower charge transfer resistance (*R*_ct_) and Li^+^ diffusion resistance (*Z*_w_) than PVDF, further indicating that DMcT improves both electron transfer kinetics and ion transport capabilities (Fig. 3i). *R*_ct_ values of PVDF and PVDF-DMcT are 101.2 Ohm and 85.1 Ohm, respectively. The above results successfully demonstrate that DMcT can not only act as an absorbing mediator to effectively confine polysulfides but also serve as a redox mediator to lower reaction energy barriers and accelerate Li_2_S conversion kinetics.

**Fig. 4:**
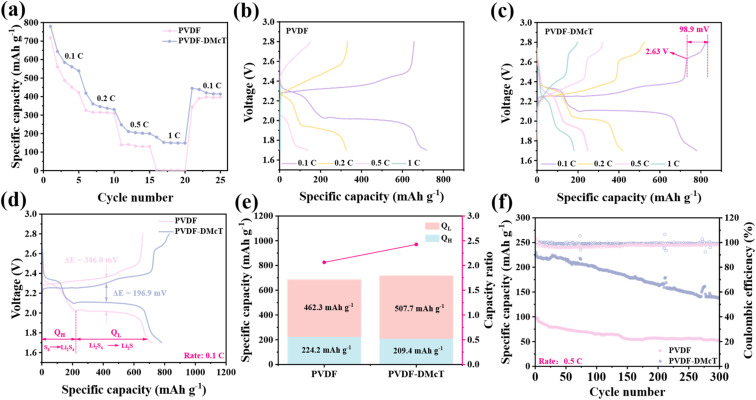
(a) Rate performances of Li–S batteries based on PVDF and PVDF-DMcT GEs from 0.1 to 1C. First-cycle charge/discharge curves of the Li–S batteries based on (b) PVDF and (c) PVDF-DMcT GEs at different current densities. (d) Comparison of the first-cycle charge/discharge curves between PVDF and PVDF-DMcT at 0.1C. (e) *Q*_L_/*Q*_H_ ratio of Li–S batteries based on PVDF and PVDF-DMcT GEs. (f) Cycling performances of Li–S batteries with PVDF and PVDF-DMcT GEs at 0.5C.

The superiority of PVDF-DMcT GE is further demonstrated by the rate performance test. As presented in Fig. 4a and S6, the battery with PVDF-20%DMcT GE exhibits a higher specific capacity, especially at high current densities (retaining 180.5 mA h g^−1^ at 1C). Upon restoration of the current density to 0.1C, the specific capacity recovers to 444.5 mA h g^−1^, underscoring its high-current durability and reversibility. Additionally, the charge/discharge features of the battery with PVDF GE nearly disappear as the current density increases to 1C (Fig. 4b and c). In contrast, the battery with PVDF-DMcT GE maintains distinct charge/discharge plateaus even at 1C, and more notably, appears to be a prolonged charge plateau at 2.63 V (98.9 mA h g^−1^), corresponding to the peak current at 2.62 V presented in Fig. 3f. These may result from the –SH groups of DMcT that generate stable Li_2_S_*n*_-DMcT complexes *via* S–H cleavage and S–S recombination.

As shown in Fig. 4d, the polarization of the Li–S battery with PVDF-DMcT GE at 0.1C is 196.9 mV, significantly lower than that of the one with PVDF GE (346 mV). This observation indicates that DMcT reduces internal resistance and enhances sulfur utilization, attributed to its increase in ionic conductivity and catalytic activity at LiPSs. The effect of DMcT on promoting sulfur conversion is further confirmed by the higher *Q*_L_/*Q*_H_ ratio in PVDF-DMcT (Fig. 4e), where *Q*_H_ and *Q*_L_ represent the kinetic energies for S_8_ → Li_2_S_4_ and Li_2_S_4_ → Li_2_S reactions, respectively. Also, cycling tests are conducted to further assess the stability of PVDF-DMcT GE. As presented in Fig. 4f, after 300 cycles, the battery with PVDF-DMcT GE retains 59.8% capacity (137.5 mA h g^−1^) at 0.5C, outperforming the one with PVDF (53.4% retention, 52.4 mA h g^−1^). The electrolyte exhibits an ultralow decay rate (0.13% per cycle) and high Coulomb efficiency (∼99.5%). As can be seen in Fig. S7a and b, compared to the PVDF cell, the PVDF-DMcT GPE cell shows a lower overpotential between the charging and discharging processes. This is because the addition of DMcT reduces the crystallinity of PVDF. In addition, the thickness of different PVDF-DMcT GE materials also has a certain impact on the electrochemical performance. During the 50 cycles at 0.1C (Fig. S8), the PVDF-DMcT cell with a thickness of 0.130 mm has the highest discharge specific capacity (813.8 mA h g^−1^). After 50 cycles, it retains a specific capacity of 619.9 mA h g^−1^, which is much higher than that of the cells with thicknesses of 0.100 mm and 0.160 mm. Moreover, the lithium ion transference of PVDF-DMcT at different thicknesses (Fig. S9) can be calculated using eqn (3). The *t*_Li^+^_ of cells with the thicknesses of 0.100 mm, 0.130 mm and 0.160 mm are 0.75, 0.84 and 0.74, respectively. This indicates the best ionic conductivity at 0.130 mm PVDF-DMcT GPs. Therefore, all the tests in the present work were conducted using 0.130 mm PVDF-DMcT.

For comparison, the lithium-sulfur gel battery fabricated by Mashekova *et al.* with a low sulfur loading (0.5 mg cm^−2^) exhibited excellent rate performance.^23^ In the present work, due to the higher sulfur loading (1.5 mg cm^−2^), the rate performance is inferior to that reported by Aiym Mashekova *et al.* However, in terms of long cycle numbers (300 cycles) at a high current (0.5C) and electrochemical impedance, the present work has a significant advantage. Similarly, in the performance test of the PVDF-Based lithium-sulfur battery conducted by Castillo *et al.*,^24^ the electrochemical performance at 0.1C button cell in the first cycle was lower than 700 mA h g^−1^, which was less than that obtained in the present work (779.6 mA h g^−1^). Besides this, the capacity retention rate over 20 cycles (less than 50%) is lower than the capacity retention rate of 59.8% achieved in the 300-cycle test described in this work. Moreover, the present work has achieved high performances under high current densities (0.5C and 1C). In addition, compared with the works of Jeong Mu Heo *et al.*,^25^ Mingjia Lu *et al.*,^26^ Tzu-Ching Chan *et al.*^27^ and Rui Li *et al.*,^28^ in terms of the long cycle performance of the PVDF-based gel electrolyte in lithium-sulfur batteries, the number of long cycles is 100, 200, 100, and 150, respectively. All of these are far less than the 300-cycle high-rate (0.5C) cycle tested in this work. Collectively, DMcT allows the PVDF GE to significantly suppress polysulfide shuttling, enhance ionic conductivity, and impart electrochemical stability.

### Mechanism

**Fig. 5:**
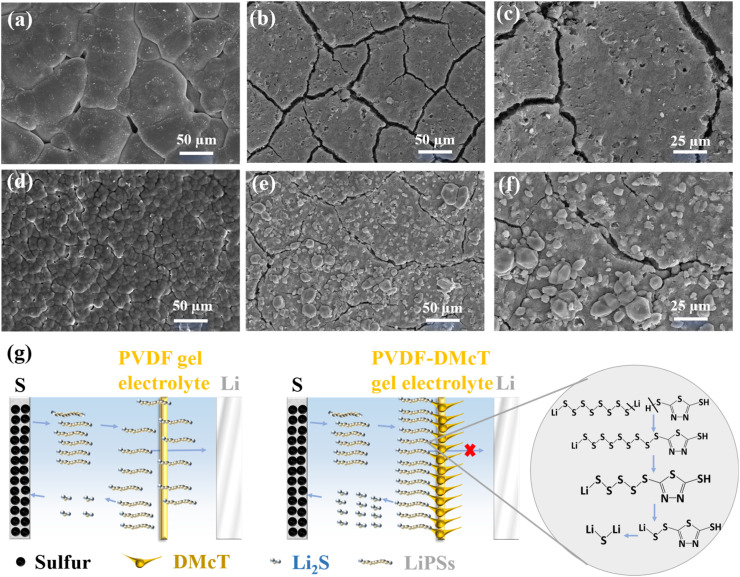
SEM images of (a) PVDF GE and (d) PVDF-DMcT GE surfaces facing the S electrode after 300 cycles at 0.5C. SEM images of the S electrode surface in contact with (b and c) PVDF GE and (e and f) PVDF-DMcT GE after 300 cycles at 0.5C. (g) Schematic depicting the underlying mechanism of PVDF-DMcT GE.

Scanning electron microscopy (SEM) was then performed to gain insight into the working mechanism of DMcT on electrochemical performance. As observed in Fig. 5a and d, distinct from the dense structure presented in PVDF GE, PVDF-DMcT GE possesses an abundant micropore structure after 300 cycles, which is conducive to suppressing the shuttling of polysulfides while offering a transport channel for Li^+^. Additionally, compared to the S electrode facing PVDF GE, the one facing PVDF-DMcT GE deposits more polysulfide particles with fewer cracks (Fig. 5b, c, e and f). This observation indicates that DMcT contributes to blocking polysulfides to prevent the loss of active substances.

Based on the above experimental results, it can be concluded that DMcT functions as a difunctional mediator to suppress the shuttle effect and accelerate the redox kinetics of Li–S batteries. This efficacy stems from the –SH groups in DMcT, which can absorb soluble polysulfides (4 ≤ *n* ≤ 8) to form stable Li_2_S_*n*_-DMcT complexes through S–H bond cleavage and recombination. As illustrated in Fig. 5g, the –SH groups of DMcT undergo cleavage to generate free radicals during the discharge process. The polysulfides (Li_2_S_*n*_, *n* = 4–8) generated by the cathodic reaction can activate S radicals in DMcT and form the DMcT-LiS_*n*_ complex. This complex will undergo chain shortening to produce DMcT-LiS, and then DMcT-LiS goes through S–S bond cleavage to regenerate DMcT. This process also promotes the conversion of Li_2_S_*n*_ to Li_2_S. Such a reversible reaction allows PVDF-DMcT GE to effectively inhibit polysulfide diffusion, catalyse lithium-ion transference, and improve ion conductivity, collectively driving the superior electrochemical performances of Li–S batteries.

## Conclusions

In summary, a novel PVDF GE for Li–S batteries is designed by taking advantage of DMcT as a difunctional mediator, which can successfully suppress polysulfide shuttling and enhance redox kinetics. The Li–S battery with optimized PVDF-DMcT GE not only demonstrates superior rate performances with a specific capacity of 180.5 mA h g^−1^ at 1C but also exhibits remarkable cycling stability with a low-capacity decay of 0.13% per cycle and a high coulombic efficiency of 99.5% over 300 cycles. The boosted performances mainly benefit from the absorbing capability of polysulfides for suppressing the shuttle effect, as well as the catalytic activity to accelerate a reversible reaction between DMcT and polysulfides (Li_2_S_*n*_, 4 ≤ *n* ≤ 8) through S–H bond cleavage and recombination. This work provides new insights into the rational design of high-performance electrolytes for Li–S batteries.

## Author contributions

X. Lin: conceptualization, data curation, formal analysis, investigation, methodology, project administration, software, validation, visualization, writing – original draft; J. Wang: methodology, formal analysis; X. Tang: methodology, formal analysis; M. Yang: data curation, formal analysis; N. Chen: conceptualization, supervision; F. Li: conceptualization, supervision, writing – review & editing; F. Zhang: conceptualization, project administration, supervision, writing – review & editing.

## Conflicts of interest

There are no conflicts to declare.

## Supplementary Material

RA-015-D5RA06093A-s001

## Data Availability

All data are contained in the manuscript and supplementary information (SI). Supplementary information is available. See DOI: https://doi.org/10.1039/d5ra06093a.

## References

[cit1] Manthiram A., Fu Y., Chung S.-H., Zu C., Su Y.-S. (2014). Chem. Rev..

[cit2] Pang Q., Liang X., Kwok C. Y., Nazar L. F. (2016). Nat. Energy.

[cit3] Peng H.-J., Huang J.-Q., Cheng X.-B., Zhang Q. (2017). Adv. Energy Mater..

[cit4] Wang J., Han W.-Q. (2022). Adv. Funct. Mater..

[cit5] Liu S., Liu X., Chen M., Wang D., Ge X., Zhang W., Wang X., Wang C., Qin T., Qin H., Qiao L., Zhang D., Ou X., Zheng W. (2022). Nano Res..

[cit6] Lu H., Zhao Y., Wang J., Liu M., Yang S., Su Y., Yuan Y. (2025). Chem. Commun..

[cit7] Hu X., Silva S. R. P., Zhang P., Liu K., Zhang S., Shao G. (2023). Chem. Eng. J..

[cit8] Zhou J., Holekevi Chandrappa M. L., Tan S., Wang S., Wu C., Nguyen H., Wang C., Liu H., Yu S., Miller Q. R. S., Hyun G., Holoubek J., Hong J., Xiao Y., Soulen C., Fan Z., Fullerton E. E., Brooks C. J., Wang C., Clément R. J., Yao Y., Hu E., Ong S. P., Liu P. (2024). Nature.

[cit9] Duan C., Cheng Z., Li W., Li F., Liu H., Yang J., Hou G., He P., Zhou H. (2022). Energy Environ. Sci..

[cit10] Cao Y., Geng C., Bai C., Peng L., Lan J., Liu J., Han J., Liu B., He Y., Kang F., Yang Q.-H., Lv W. (2025). Energy Environ. Sci..

[cit11] Wu X., Pan H., Zhang M., Zhong H., Zhang Z., Li W., Sun X., Mu X., Tang S., He P., Zhou H. (2024). Advanced Science.

[cit12] Wang C., Kim J. T., Wang C., Sun X. (2023). Adv. Mater..

[cit13] Yu P., Sun S., Sun C., Zeng C., Hua Z., Ahmad N., Shao R., Yang W. (2024). Adv. Funct. Mater..

[cit14] Wei Z., Ren Y., Sokolowski J., Zhu X., Wu G. (2020). InfoMat.

[cit15] Yan W., Wei J., Chen T., Duan L., Wang L., Xue X., Chen R., Kong W., Lin H., Li C., Jin Z. (2021). Nano Energy.

[cit16] Chen D., Zhu M., Kang P., Zhu T., Yuan H., Lan J., Yang X., Sui G. (2022). Advanced Science.

[cit17] Shen Y.-Q., Zeng F.-L., Zhou X.-Y., Wang A.-b., Wang W.-k., Yuan N.-Y., Ding J.-N. (2020). J. Energy Chem..

[cit18] Pei F., Dai S., Guo B., Xie H., Zhao C., Cui J., Fang X., Chen C., Zheng N. (2021). Energy Environ. Sci..

[cit19] Xia Y., Wang X., Xia X., Xu R., Zhang S., Wu J., Liang Y., Gu C., Tu J. (2017). Chem.–Eur. J..

[cit20] Jana S., Garain S., Sen S., Mandal D. (2015). Phys. Chem. Chem. Phys..

[cit21] Wang Z., Feng L., Deng C., Wang S. (2022). Chem. Eng. J..

[cit22] Jin Z.-Q., Liu Y.-G., Wang W.-K., Wang A.-B., Hu B.-W., Shen M., Gao T., Zhao P.-C., Yang Y.-S. (2018). Energy Storage Mater..

[cit23] Mashekova A., Umirzakov A., Yegamkulov M., Aliyakbarova M., Uzakbaiuly B., Nurpeissova A., Bakenov Z., Mukanova A. (2025). RSC Adv..

[cit24] Castillo J., Robles-Fernandez A., Cid R., González-Marcos J. A., Armand M., Carriazo D., Zhang H., Santiago A. (2023). Gels.

[cit25] Heo J. M., Mun J., Lee K. H. (2024). Macromol. Res..

[cit26] Lu M., Chen K., Jia Z., Ren J., He P., Yang S., Bagherzadeh R., Lai F., Miao Y.-E., Liu T. (2024). Energy Storage Mater..

[cit27] Chan T.-C., Chung S.-H. (2024). ACS Sustain. Chem. Eng..

[cit28] Li R., Chen Q., Jian J., Hou Y., Liu Y., Liu J., Xie H., Zhu J. (2024). J. Power Sources.

