# Supplementary material for: 2,5-Dimercapto-1,3,4-thiadiazole-modified gel electrolyte for reduced shuttle effect and enhanced redox kinetics of lithium–sulfur batteries
Source: RSC Adv. 2025 Oct 27;15(48):41148–55. doi: 10.1039/d5ra06093a (PMC12558135; doi:10.1039/d5ra06093a)
Supplement: RA-015-D5RA06093A-s001 [file RA-015-D5RA06093A-s001.pdf]

Supplementary Information (SI) for RSC Advances.
This journal is © The Royal Society of Chemistry 2025

Supplementary Materials for

2,5-dimercapto-1,3,4-thiadiazole Modified Gel Electrolyte for Reduced Shuttle Effect and Enhanced Redox Kinetics of Lithium–Sulfur Batteries

Xiangzhe Lin,[a] Junlin Wang,[a] Xu Tang,[b] Manru Yang,[c] Nairong Chen,[c] Feng Li*[c] and Fengxiang Zhang*[b]

[a]Leicester International Institute, Dalian University of Technology, Dalian 124221, China. Email: linxiangzhe25fz@163.com

[b]School of Chemical Engineering, Ocean and Life Sciences (State Key Laboratory of Fine Chemicals), Dalian University of Technology, Dalian 124221, China. Email: zhangfx@dlut.edu.cn

[c]College of Material Engineering, Fujian Agriculture and Forestry University, Fuzhou, 350002, China. Email: fengli@fafu.edu.cn

*Corresponding authors: fengli@fafu.edu.cn; zhangfx@dlut.edu.cn

Supplementary figures

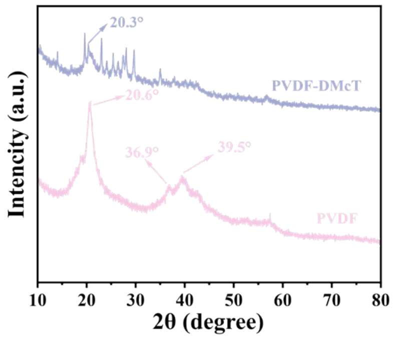

Fig. S1 XRD patterns of PVDF and PVDF-DMcT samples.

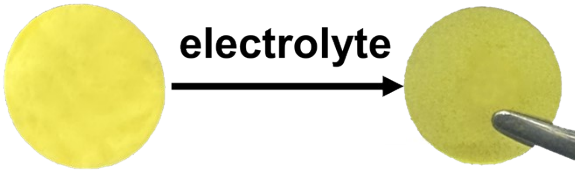

Fig. S2 Photos of PVDF-DMcT GEP before and after absorbing LiTFSI.

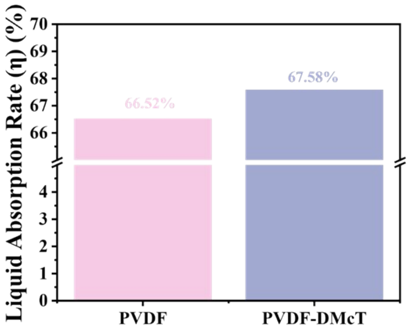

Fig. S3 Liquid absorption rate of PVDF GE and PVDF-DMcT GE.

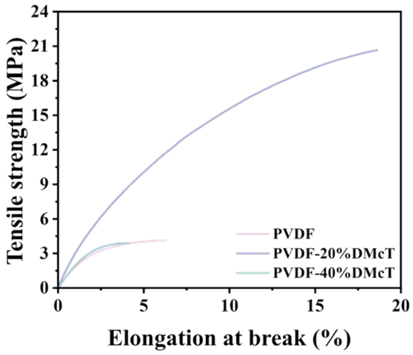

Fig. S4 The tensile strength-elongation at break curves of PVDF, PVDF-20%DMcT, and PVDF-40%DMcT films.

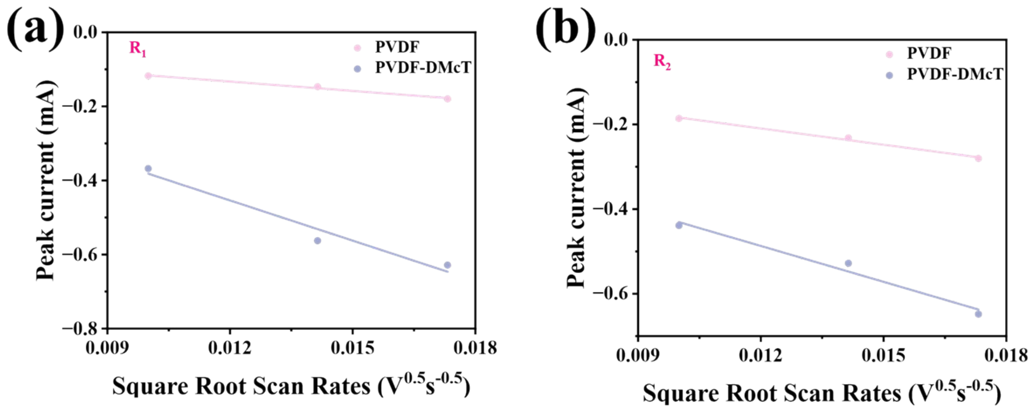

Fig. S5 Fitting plot of peak current at the (a) R_1 and (b) R_2 peaks versus the square root of scan rate.

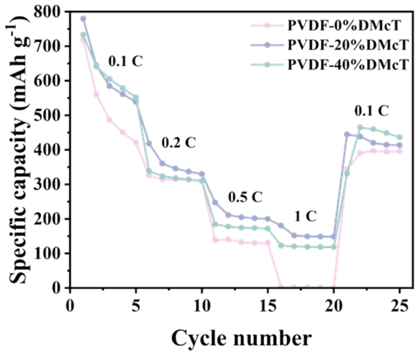

Fig. S6 Rate performances of the Li-S batteries based on PVDF and PVDF-DMcT GE from 0.1 to 1 C.

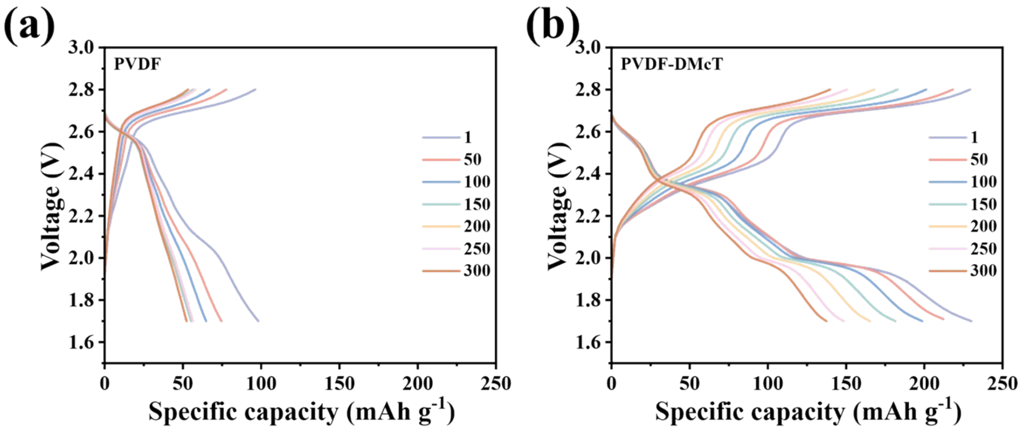

Fig. S7 Charge/Discharge profiles of Li-S cells based on PVDF and PVDF-DMcT GEs at different cycles at 0.5 C.

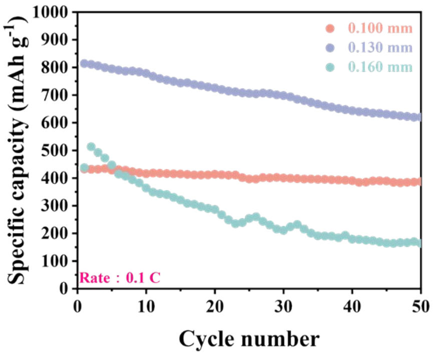

Fig. S8 Cycling performances of Li-S batteries with different PVDF-DMcT GE thicknesses.

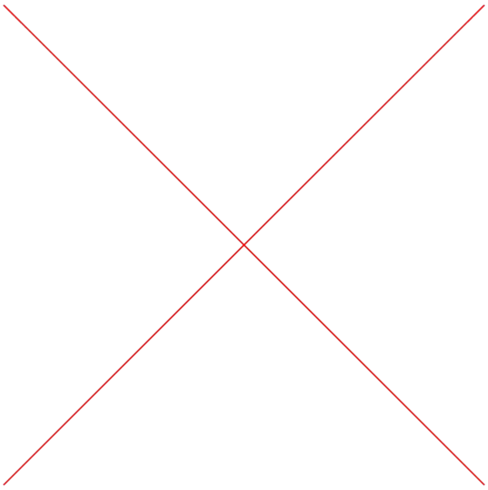

Fig. S9 Lithium ions transference number with different PVDF-DMcT GE thicknesses.

Table S1. The battery performance under the similar sulfur loading.

	**The present work**	**Julen Castillo et al.** [1]	**Jeong Mu Heo et al.** [2]	**Mingjia Lu et al.** [3]	**Rui Li et al.** [4]	**Yan Xia et al.** [5]	**Longyan Li et al.** [6]
**S loading ($mg\ cm^{-2}$)**	**1.5**	**1.5**	**Not indicated**	**1**	**1.5**	**1.7**	**1.8**
**Cycles**	**300**	**20**	**100**	**200**	**150**	**40**	**50**
**0.1 C Discharge capacity (mAh/g)**	**813.8**	**~680**	**899**	**Not indicated**	**~780**	**744.3**	**675**

Reference:

1. J. Castillo, A. Robles-Fernandez, R. Cid, J. A. González-Marcos, M. Armand, D. Carriazo, H. Zhang and A. Santiago, *Gels*, 2023, **9**, 336.
2. J. M. Heo, J. Mun and K. H. Lee, *Macromolecular Research*, 2024, **32**, 187-196.
3. M. Lu, K. Chen, Z. Jia, J. Ren, P. He, S. Yang, R. Bagherzadeh, F. Lai, Y.-E. Miao and T. Liu, *Energy Storage Materials*, 2024, **73**, 103870.
4. R. Li, Q. Chen, J. Jian, Y. Hou, Y. Liu, J. Liu, H. Xie and J. Zhu, *Journal of Power Sources*, 2024, **624**, 235521.
5. Y. Xia, X. Wang, X. Xia, R. Xu, S. Zhang, J. Wu, Y. Liang, C. Gu and J. Tu, *Chemistry – A European Journal*, 2017, **23**, 15203-15209.
6. L. Li, Y. Chen, X. Guo and B. Zhong, *Polymer Chemistry*, 2015, **6**, 1619-1626.
